# Superfinishing with Abrasive Films Featuring Discontinuous Surfaces

**DOI:** 10.3390/ma17071704

**Published:** 2024-04-08

**Authors:** Katarzyna Tandecka, Wojciech Kacalak, Maciej Wiliński, Michał Wieczorowski, Thomas G. Mathia

**Affiliations:** 1 Department of Engineering and Informatics Systems, Faculty of Mechanical Engineering and Energy, Koszalin University of Technology, 75620 Koszalin, Poland; wojciech.kacalak@tu.koszalin.pl; 2 Independent Reasercher, 75412 Koszalin, Poland; wilinskimaciej@wp.pl; 3 Faculty of Mechanical Engineering, Institute of Applied Mechanics, Poznan University of Technology, 3 Piotrowo St., 60965 Poznan, Poland; michal.wieczorowski@put.poznan.pl; 4 Laboratoire de Tribologie et Dynamique des Systemes (LTDS), Ecole Centrale de Lyon, Centre National de la Recherche Scientifique, 69134 Lyon, France; thomas.mathia@ec-lyon.fr

**Keywords:** surface finishing, abrasive film, finishing, abrasion, superfinishing, discontinuous surface

## Abstract

This study introduces innovative designs for abrasive tools aimed at enhancing surface finishing processes. Prototypes consisting of non-continuous abrasive films with discontinuous surface carriers and abrasive layers were developed to improve the efficiency and effectiveness of the smoothing process. Four distinct abrasive films with varying nominal grain sizes were fabricated to explore the versatility and efficacy of the prototypes. The results indicate that the incorporation of carrier irregularities significantly influences surface finishing processes, leading to improvements in material removal efficiency and surface quality. Longitudinal discontinuities facilitate faster removal of irregularities from workpiece materials, reducing the risk of deep scratches on surfaces. Additionally, this study highlights the importance of tool motion patterns in optimizing material removal processes and ensuring surface quality. The integration of carrier irregularities with additional oscillatory tool motion shows promise for further improving surface quality. These findings advance our understanding of abrasive machining processes and provide valuable insights for optimizing abrasive tool designs and machining strategies for enhanced surface finishing.

## 1. Introduction

**Figure 1:**
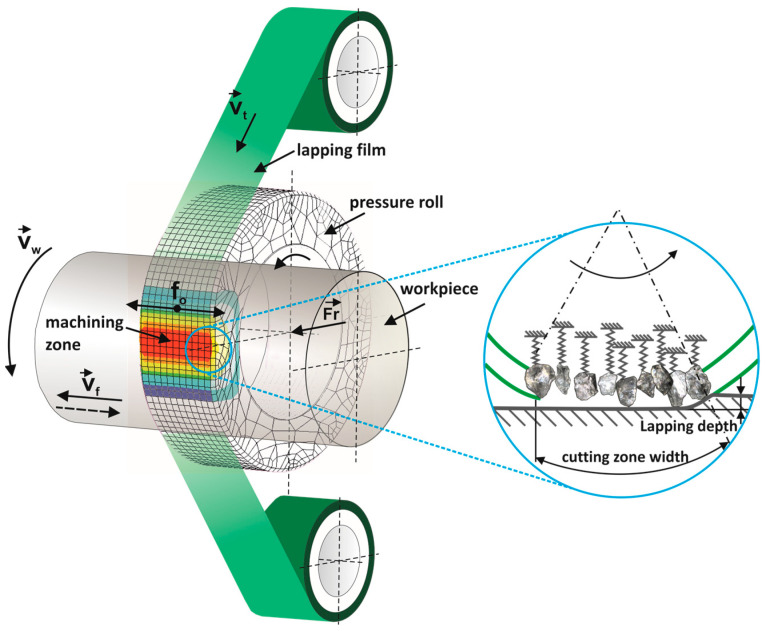
Kinematic diagram of rotary surface finishing using lapping film, where the following quantities are indicated on the diagram: *v_t_*—tool speed, *v_w_*—workpiece speed, *v_f_*—tool feed speed, *f_o_*—tool oscillation frequency, and *F_r_*—the pressure force of the pressing roller [18].

Abrasive films are used in a surface finishing method distinguished by the fact that the tool is only used once. Therefore, it is essential to design the tools in such a way as to optimize them [1,2]. The tool moves very slowly compared to the speed of the workpiece being processed [3]. The new film is unwound, introduced into the machining zone, and pressed against the workpiece by a pressure roller [4,5,6]. It is then withdrawn from the machining zone and wound onto a take-up roller (Figure 1). The tool moves over the workpiece with an applied feeding motion, and additionally, an oscillatory motion of the tool can be applied to enhance the surface smoothing effect [7,8,9]. This method ensures precise control over the surface finishing process, allowing for consistent and high-quality results. Additionally, the single-use nature of abrasive films reduces the risk of contamination and ensures reliability in achieving desired surface characteristics [10,11].

The utilization of abrasive films in industrial applications for surface smoothing has garnered significant attention due to its efficiency and reliability. This method offers a controlled and precise approach to achieving desired surface characteristics while minimizing the risk of contamination. By employing abrasive films, manufacturers can optimize their processes by ensuring consistent and high-quality surface finishes [12,13,14]. The single-use nature of abrasive films further enhances reliability, as it eliminates the potential for cross-contamination and ensures that each surface finishing operation is performed with a fresh tool [15,16]. A microfinishing attachment is easy to implement in industrial settings because it can be mounted on conventional machining equipment, facilitating the adoption of this technology [17]. This ease of integration enables manufacturers to enhance their existing machinery with surface smoothing capabilities without significant modifications. Additionally, the straightforward installation process minimizes downtime and the disruption of production operations. Overall, the accessibility of microfinishing attachments simplifies the adoption of surface smoothing techniques in industrial settings, promoting efficiency and productivity.

Abrasive films have diverse applications across critical industries where surface precision is paramount. In the automotive sector [19], they are employed to polish engine components, transmission parts, and crankshafts, optimizing their performance and durability [20]. The aerospace industry utilizes abrasive films for refining turbine blades, aircraft engine components, and landing gear parts, ensuring both aerodynamic efficiency and structural integrity. In the medical field, this technology is instrumental in finishing surgical implants, prosthetic joints, and orthopedic instruments to meet stringent quality and biocompatibility standards. Furthermore, abrasive films are extensively utilized in the manufacturing of precision bearings, gears, and shafts in the machinery industry, thereby enhancing their operational efficiency and longevity [21]. The marine sector benefits from the application of abrasive films in the polishing of ship propellers, hull components, and marine engine parts to improve hydrodynamics and reduce fuel consumption. Moreover, they are indispensable in the electronics industry for refining semiconductor components, printed circuit boards, and precision electronic devices to ensure optimal functionality and reliability. Additionally, in the optics industry, abrasive films play a crucial role in finishing lenses, mirrors, and optical fibers to achieve superior optical performance and clarity. Lastly, their application in the jewelry industry facilitates the polishing of precious metals, gemstones, and intricate jewelry designs, elevating their aesthetic appeal and commercial value.

**Figure 2:**
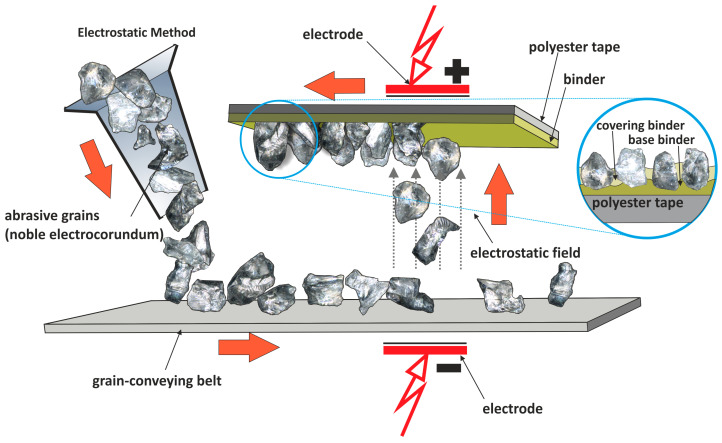
The production scheme in the electrostatic field of a microfinishing film.

**Figure 3:**
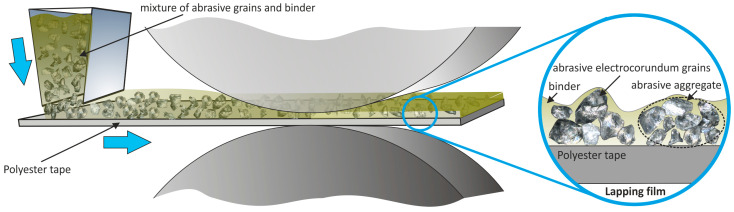
Schematic depicting the production of lapping film [18].

There are two main types of abrasive films utilized in surface finishing processes: microfinishing films (Figure 2) and lapping films (Figure 3). Microfinishing films are designed for microfinishing applications, where abrasive grains are embedded in a polymeric matrix using an electrostatic field [22,23], resulting in sharper cutting edges [24,25,26]. These films are ideal for achieving precise surface finishes and are commonly used in the automotive, aerospace, and medical industries [27,28]. This type of abrasive grain is commonly used in endless belts [29,30,31]. On the other hand, lapping films feature abrasive grains fully immersed in a binder, making them suitable for superfinishing applications. In these films, the abrasive particles are evenly distributed throughout the binder, ensuring consistent material removal and surface refinement [32]. Lapping films are widely used in superfinishing processes where the goal is to achieve extremely smooth and flat surfaces with minimal roughness and waviness [23,33]. They are commonly employed in industries like semiconductor manufacturing, where the quality of surface finishes directly impacts device performance and reliability.

The quest for superior surface finishing techniques has led to innovative approaches in abrasive tool design [34,35,36,37]. In this study, we introduce a novel concept focusing on the development of abrasive tool prototypes comprising non-continuous abrasive films with a unique discontinuous surface carrier and an abrasive layer. The primary objective is to revolutionize the efficiency and effectiveness of surface finishing processes. Traditionally, surface finishing involves sequential applications of abrasive materials. However, our method deviates significantly by integrating discontinuous features into the abrasive tools. To evaluate the versatility and efficacy of our prototypes, we fabricated distinct lapping films with varying nominal grain sizes, ranging from 30 to 5 μm. Due to the high precision requirements imposed on lapping film tools, we decided to investigate the discontinuities of this type of tool, as loose abrasives can have a detrimental effect, especially in such highly precise machining processes.

Abrasive films are versatile tools that can be used to machine a wide range of materials across various industries. Common materials processed using abrasive films include metals such as steel, aluminum, and titanium, which are frequently encountered in automotive, aerospace, and machinery manufacturing [38]. Additionally, abrasive films are employed in the finishing of composite materials like carbon fiber and fiberglass, which are used extensively in the aerospace, marine, and sporting goods industries. They are also suitable for processing ceramics, including alumina, zirconia, and silicon carbide, which are commonly found in electronic components and medical implants [39]. Moreover, abrasive films can be utilized for finishing plastics and polymers, such as acrylics, polycarbonates, and polyesters, which are used in diverse applications ranging from consumer electronics to medical devices. Overall, abrasive films offer a versatile solution for achieving precise surface finishes across a broad spectrum of materials, making them indispensable in modern manufacturing processes.

Our approach involves two types of abrasive films characterized by different patterns of discontinuities on their surfaces. The first type is designed for finishing without tool oscillation, focusing on creating outlets for loose particles through the tool’s surface discontinuities. The second type incorporates tool oscillation, utilizing the inherent properties of abrasive materials to achieve a refined surface finish. Through detailed analysis and experimentation, we explore the impact of these discontinuous features on the surface finishing process. Utilizing advanced microscopy techniques, we examine the worn surfaces of abrasive films and evaluate their effectiveness in mitigating surface imperfections. Furthermore, we conduct comprehensive research on the topography of finished surfaces, comparing the results obtained with conventional abrasive tools to those achieved with our innovative prototypes. The analysis reveals significant improvements in surface quality and material removal efficiency with the use of discontinuous abrasive tools. Overall, our study presents a paradigm shift in surface finishing methodologies, highlighting the potential for discontinuous abrasive tools to enhance efficiency, reduce material consumption, and improve surface quality. The findings offer valuable insights for future abrasive tool designs and machining strategies, paving the way for advancements in surface engineering technologies. 

Our study addresses a gap in the existing research by introducing a novel concept focusing on the development of abrasive tool prototypes comprising non-continuous abrasive films with a unique discontinuous surface carrier and an abrasive layer. This innovative approach deviates significantly from traditional surface finishing methods. Our study aims to significantly enhance the efficiency and effectiveness of surface finishing processes by introducing a novel concept. The uniqueness of our approach lies in the utilization of irregularities in the carriers of lapping films, which has not been explored or proposed by any other researchers worldwide. This novel solution was validated through a thorough patent purity study [4], highlighting its originality and unprecedented nature in the field of surface engineering technologies. Therefore, our study not only fills a gap in the existing research by proposing a novel solution to surface finishing but also advances the understanding and application of abrasive tool design methodologies.

## 2. Materials and Methods

### 2.1. Lapping Films with Discontinuous Surfaces

**Figure 4:**
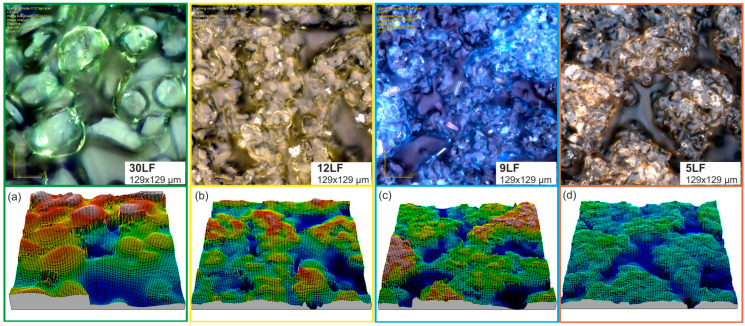
Images of new lapping films after data acquisition with an Olympus LEXT OLS4000 confocal microscope (Tokyo, Japan), where 30LF denotes a lapping film with an abrasive grain size of 30 μm (**a**), 12LF denotes a lapping film with an abrasive grain size of 12 μm (**b**), 9LF denotes a lapping film with an abrasive grain size of 9 μm (**c**), and 5LF denotes a lapping film with an abrasive grain size of 5 μm (**d**). The measurement fields are 129 × 129 μm.

In this study, we aimed to innovate the design of abrasive tools for surface smoothing processes. Our approach involved the development of prototypes consisting of non-continuous abrasive films characterized by a discontinuous surface carrier and an abrasive layer. This novel design was conceived to enhance the efficiency and effectiveness of the smoothing process. The methodology employed the sequential application of lapping film to achieve the desired surface finish. To explore the versatility and efficacy of our prototypes, we fabricated four distinct abrasive films with varying nominal grain sizes. These included prototypes with nominal grain sizes of 30 μm (30LF) (Figure 4a), 12 μm (12LF) (Figure 4b), 9 μm (9LF) (Figure 4c), and 5 μm (5LF) (Figure 4d). The development of these abrasive tool prototypes involved a significant departure from conventional designs. The developed solutions were patented, and a patent was obtained. Details regarding this patent are provided in Section 5 of this article.

**Figure 5:**
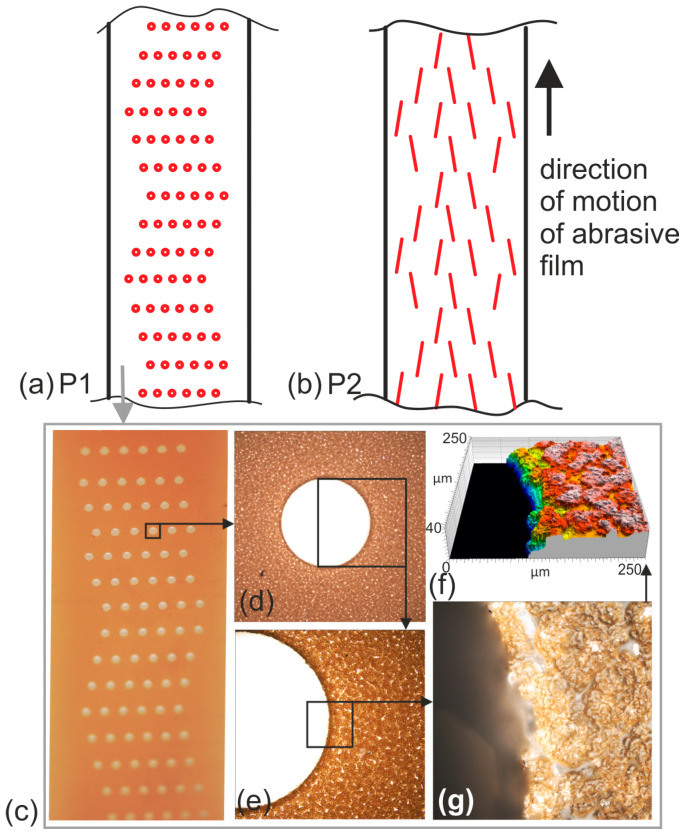
Schematics of discontinuity patterns on the surfaces of abrasive tools designed for microfinishing without the use of tool oscillation (P1) (**a**) and for application during finishing with tool oscillation (P2) (**b**). Image of the film with pattern P1 (**c**) as well as confocal microscope images of the P1 prototype (**d**,**e**,**g**) and a map of the height of the discontinuity edges of P1 (**f**).

Two types of abrasive films characterized by different patterns of discontinuities on their surfaces were designed and fabricated. The first type of abrasive film was designed for smoothing without the application of tool oscillation, thus eliminating the need to introduce additional movement of abrasive grains into the machining zone. Instead, the focus was on creating spaces that would serve as outlets for loose particles, including fragmented abrasive grains, from the machining zone. The discontinuity on the tool’s surface was created by employing multiple perforations along the entire length of the tool in a specific pattern (Figure 5a). The tool prototypes were developed by modifying conventional tools. The discontinuities were cut through the outlet, penetrating both the abrasive layer and the polyester backing (Figure 5c–f). The second type of tool (Figure 5b) was meticulously crafted for smoothing while incorporating tool oscillation. Its conceptualization hinged on the premise that abrasive grains surrounding longitudinal discontinuities, which are inclined relative to the tool’s direction of movement, would instigate supplementary micro-oscillations in those regions. This strategic integration aimed to harness the inherent properties of abrasive materials to achieve a more refined surface finish. By deliberately introducing these discontinuities along the length of the tool in a specific configuration, we sought to exploit the dynamic interaction between the abrasive grains and the workpiece surface. This innovative approach was envisaged to not only enhance the efficiency of the smoothing process but also to impart a higher degree of control over surface texture.

### 2.2. Superfinishing Process

**Figure 6:**
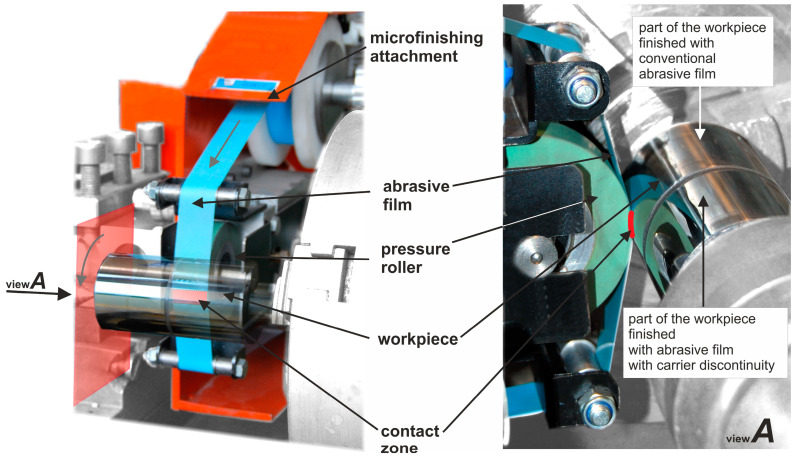
Research setup for the surface finishing process using abrasive films with discontinuous surfaces.

Research on the finishing process using abrasive films with applied carrier discontinuity and a microfinishing attachment (Figure 6) was conducted employing a pressure roller with a hardness of 50 °Sh A. The roller force applied to the workpiece was 50 N, the speed of the abrasive film was 160 mm/min, and the peripheral speed of the workpiece was 40 m/min (Table 1). The total duration of the microfinishing process was 440 s, with a single operation processing time of 110 s. Sequential microfinishing process investigations using the abrasive films LF30, LF12, LF9, and LF5 were conducted on samples of chrome steel (40H) heat-treated to a hardness of 60 HRC. The samples were divided into two parts (Figure 6), the first of which was processed with an abrasive film with applied carrier discontinuity, while the second part was smoothed with conventional abrasive film, ensuring identical processing conditions to guarantee high-quality comparative research results. The finishing of the surfaces was carried out using reciprocating motion, meaning the workpiece and the abrasive film moved in opposite directions relative to each other. Additionally, a feeding motion was also applied to the workpiece. The frequency of oscillation during smoothing with abrasive film with surface irregularity pattern P2 was 300 cycles per minute, and the oscillation amplitude was A = 2.5 mm.

### 2.3. Research on the Topography of Finished Surfaces

**Figure 7:**
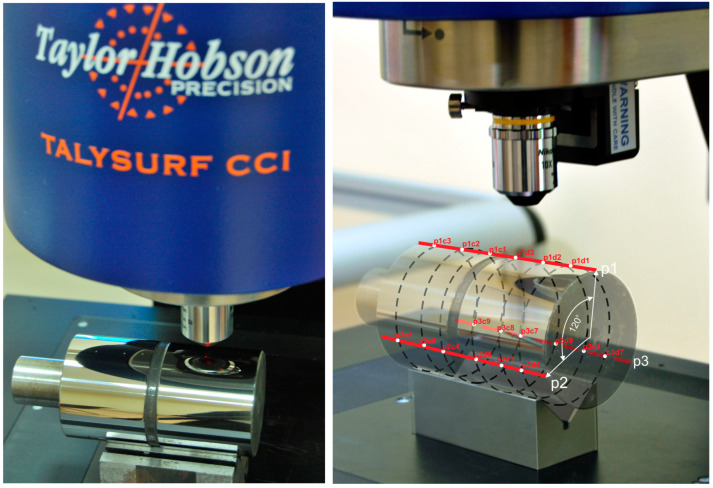
Measurement setup of the processed surfaces, with measurement points marked on the workpiece being processed.

In each prepared sample zone, nine measurement points of the geometric structures of the processed surfaces were determined, for a total of 18 measurement points after each smoothing procedure. Nine of the points were on the surface smoothed with abrasive film with a discontinuous surface, and the other nine were on the surface smoothed with a conventional tool (Figure 7). Measurements of the geometric structures at all measurement points were carried out using a Talysurf CCI6000 measurement system from Taylor Hobson (Leicester, UK). A 50× magnification objective was employed, providing a measurement field of 0.36 × 0.36 mm. The acquired outcomes were averaged for analysis.

### 2.4. Analysis of Surface Irregularities on the Used Abrasive Film

In order to analyze the surfaces of the lapping films after the finishing process and the worn tool surfaces around the discontinuities of the carrier, a Phenom ProX (Thermo Fisher Scientific, Waltham, MA, USA) tabletop electron microscope was utilized. This SEM microscope (Phenom-World BV, Eindhoven, The Netherlands) enabled the observation of the machining products on the surfaces of the abrasive films.

## 3. Results and Discussion

### 3.1. SEM Analysis of Finished Products

**Figure 8:**
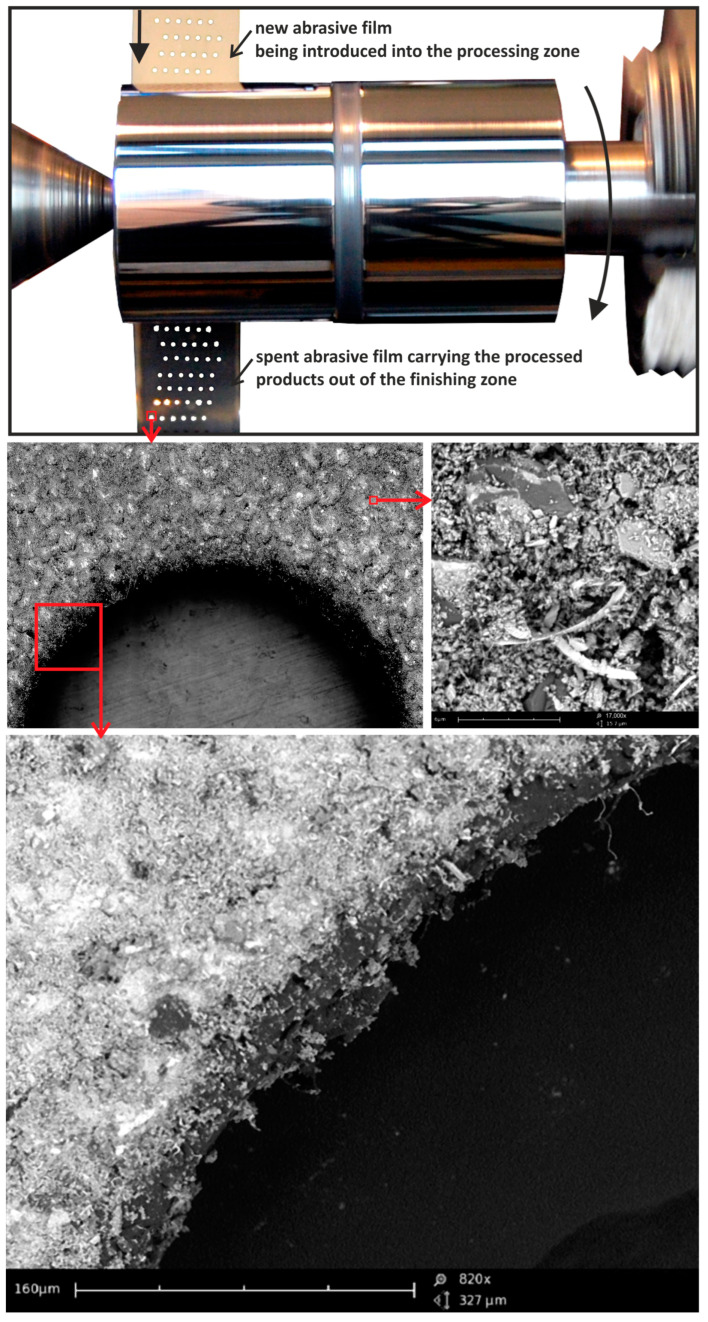
SEM images of the spent surface of an abrasive film with pattern P1 after processing.

After the sequential finishing process using abrasive films with nominal grain sizes ranging from 30 to 5 μm, the worn tools were examined. Initially, the abrasive films with a pattern of non-continuous features (referred to as P1) were analyzed. This pattern comprised a series of holes on the tool surfaces. The processing was carried out with the tool moving in a feeding motion. As anticipated, longitudinal chips were observed on the edges of the abrasive film’s discontinuities (Figure 8). In a conventional process, these chips would typically be fragmented within the spaces between grains, indicating that the discontinuities in the form of holes on the tool surfaces could serve as excellent pathways for the removal of larger particles. This finding is significant, as it suggests that such discontinuities may effectively mitigate the negative impacts of larger particles on machined surfaces.

**Figure 9:**
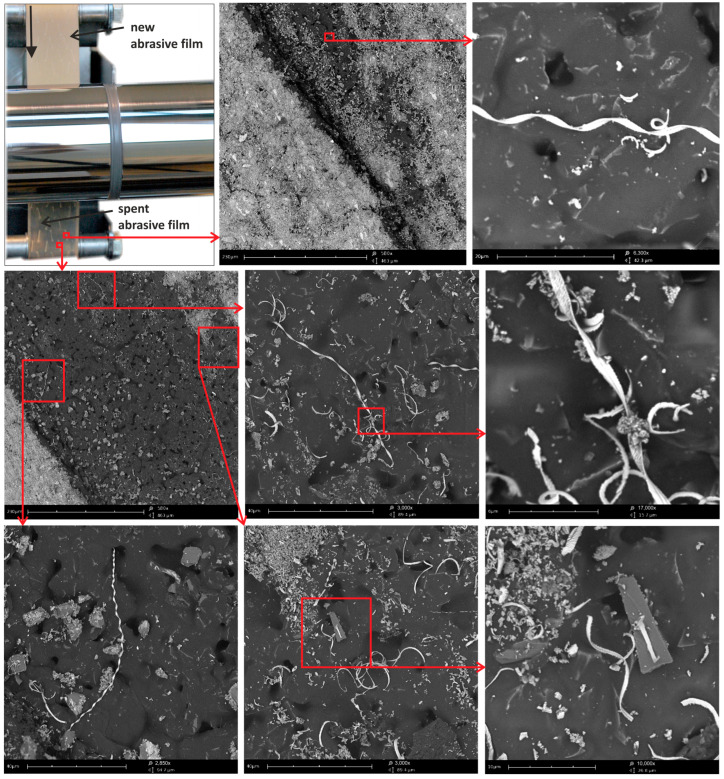
SEM images of the spent surface of the abrasive film with pattern P after processing.

Furthermore, the examination revealed the effectiveness of the tool’s feeding motion in facilitating the removal of larger particles through the discontinuities. This observation underscores the importance of a tool’s movement pattern in optimizing the material removal process and ensuring surface quality. Moreover, the presence of longitudinal chips specifically at the edges of the abrasive film’s discontinuities highlights the role of these features as preferential channels for chip evacuation. This insight could inform the design of future abrasive tools to strategically incorporate such discontinuities for enhanced material removal efficiency. The worn surfaces of abrasive films with the P2 irregularity pattern were also examined. These irregularities consisted of longitudinal deviations from the direction of film movement, and the smoothing process was carried out with additional oscillatory motion of the tool. In the depressions of the tool’s irregularities, large quantities of loose abrasive grains were observed (Figure 9), indicating that all these abrasive grains were torn from the binder and were completely uncontrolled in the machining zone, which could have a very negative effect on the formation of individual deep scratches on the machined surface. It is worth mentioning that abrasive grains embedded and bound in the binder penetrate into the workpiece material to a maximum of 10% of their nominal size [18]. However, such loose grains can cause the formation of much deeper scratches [40,41]. From this perspective, irregularities on the tool in this machining process are crucial due to the removal of loose particles. Additionally, very long ribbon-shaped chips, which remained intact and were not fragmented, could also be observed in the irregularities on the stored film, further confirming that excess machining products can be removed through irregularities in the carrier. This examination further highlights the critical role of tool motion patterns in optimizing material removal processes and ensuring surface quality. Specifically, the presence of longitudinal chips at the edges of abrasive film discontinuities underscores their significance as preferential pathways for chip evacuation. This finding not only emphasizes the importance of strategically incorporating such discontinuities in future abrasive tool designs but also suggests potential avenues for enhancing material removal efficiency. Moreover, the investigation into the P2 irregularity pattern on worn abrasive films unveiled additional complexities in material removal dynamics and surface quality during the smoothing process. The presence of significant quantities of loose abrasive grains within the tool’s irregularities underscores the challenges posed by uncontrolled particle distributions in machining operations. The unregulated presence of loose grains can significantly compromise surface integrity, leading to the formation of deep scratches and surface imperfections. From this perspective, the strategic management of the irregularities on a tool’s surface becomes paramount in mitigating the adverse effects of loose particles. Additionally, the presence of long, intact, ribbon-shaped chips within the irregularities further underscores the role of these features as efficient channels for removing excess machining products. This observation reinforces the notion that irregularities in the carrier medium play a crucial role in regulating debris evacuation, ultimately contributing to improved surface quality.

### 3.2. Research on Finished Surfaces

The surfaces were examined after the micro-smoothing processes using tools featuring carrier irregularities and conventional machining equipment. Using the ISO 25178 standard [42], the following height parameters were determined for surface description:

- Sp: maximum height of peaks;
- Sv: maximum height of valleys;
- Sz: maximum height of the surface;
- Sa: arithmetical mean height of the surface.

**Figure 10:**
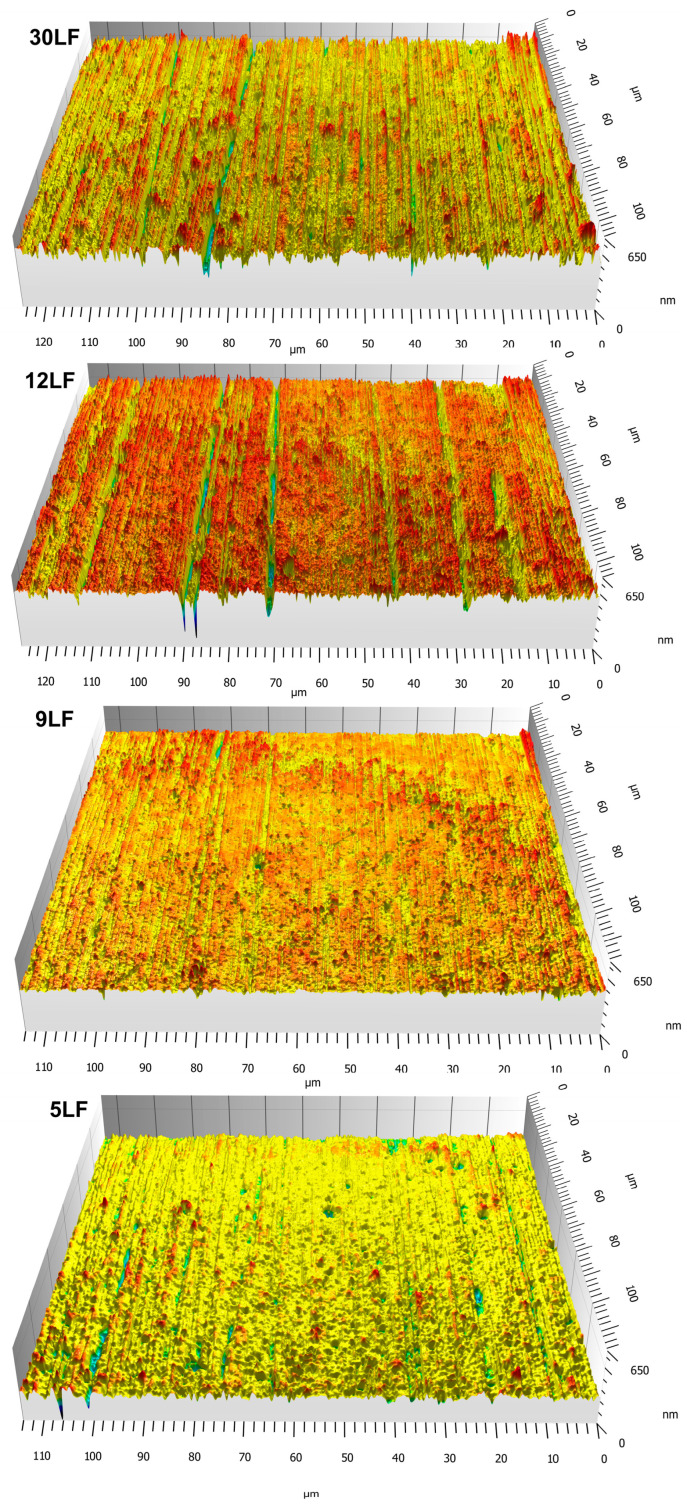
The surfaces of the workpiece after the sequential finishing process using consecutive abrasive films with nominal grain sizes ranging from 30 to 5 μm.

In Figure 10, selected sections of the measured surfaces are depicted after the finishing process using conventional abrasive tools with tool oscillation motion. After each machining operation, the surfaces were measured at nine points. Thus, throughout the entire research process, the parameters for assessing surface roughness were determined for 180 measurement surfaces.

**Figure 11:**
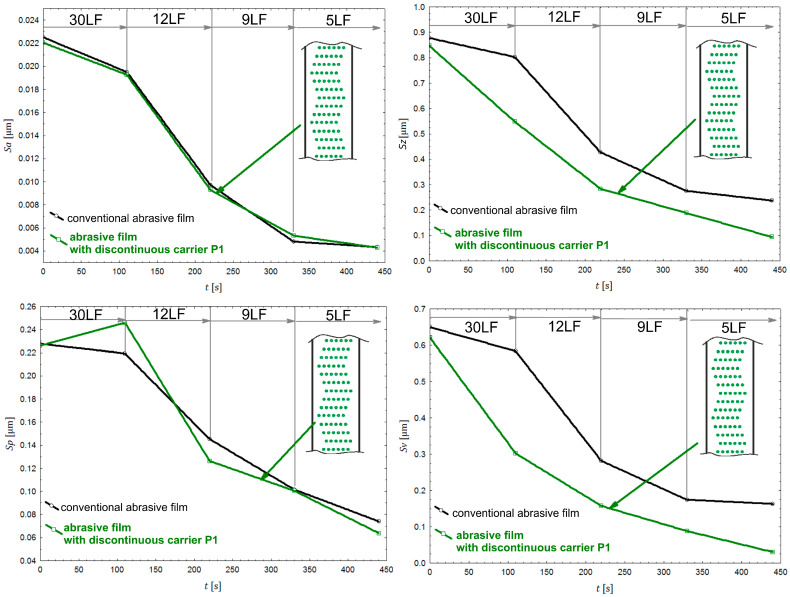
The parameters (Sa, Sz, Sp, and Sv) for assessing surface roughness after the finishing process with abrasive films featuring P1 carrier irregularities (green color) and conventional abrasive films with nominal grain sizes in micrometers: 30 (30LF), 12 (12LF), 9 (9LF), and 5 (5LF).

When analyzing the parameter values for assessing surface roughness after the finishing process with abrasive films featuring carrier irregularities in pattern P1 (Figure 11), focusing solely on the Sa parameter, i.e., the arithmetical mean height of the surface, the results using the modified film and the conventional film were practically the same. 

This implies that on films with significantly fewer abrasive grains, the results remained the same. In this specific case, it means that on an abrasive film with 14% fewer abrasive grains than the conventional film, the results remained consistent. This has significant implications globally for the consumption of materials in the production of abrasive films. Using both conventional abrasive film and abrasive film with carrier irregularity resulted in similar outcomes. In both cases, the same tool pressure force was applied to the workpiece, resulting in the same width of the tool–workpiece contact zone. For the innovative tools, the introduction of irregularities to the contact zone led to fewer abrasive grains, thus increasing the pressure force applied to individual grains. This could explain the similarity in the results. Certainly, during the surface finishing process, the sizes of abrasive grains, the way they are deposited on the film, and their concentration are crucial in analyzing the effectiveness of surface finishing. However, research on surface finishing with carrier irregularities, where there are 14% fewer abrasive grains, also indicates that the manner and amount of accumulation of machining products and abrasive grains torn from the binder between the abrasive grains play a crucial role in creating high-quality smoothed surfaces. Introducing irregularities that effectively remove large loose particles from the machining zone is of paramount importance. 

On the other hand, the analysis of the Sv parameter, i.e., the maximum height of valleys, confirmed the theory that uncontrolled loose abrasive particles torn from the tool surface can lead to the formation of deeper scratches through carrier irregularities. After treatment with abrasive films with a nominal grain size of 30, significantly shallower scratches were achieved on the surface with the film with carrier irregularity (depth of 0.3 μm) compared to the conventional film (depth of 0.6 μm). Comparable results were achieved after machining with a conventional film with a nominal grain size of 12 μm and after using the 30LF abrasive film with the P1 irregularity. This demonstrates the effectiveness of utilizing abrasive films with carrier irregularities to reduce scratch depth and highlights their potential to improve surface quality. Additionally, it underscores the importance of considering the influence of carrier irregularities in optimizing abrasive film selection and machining processes for enhanced surface finishing.

**Figure 12:**
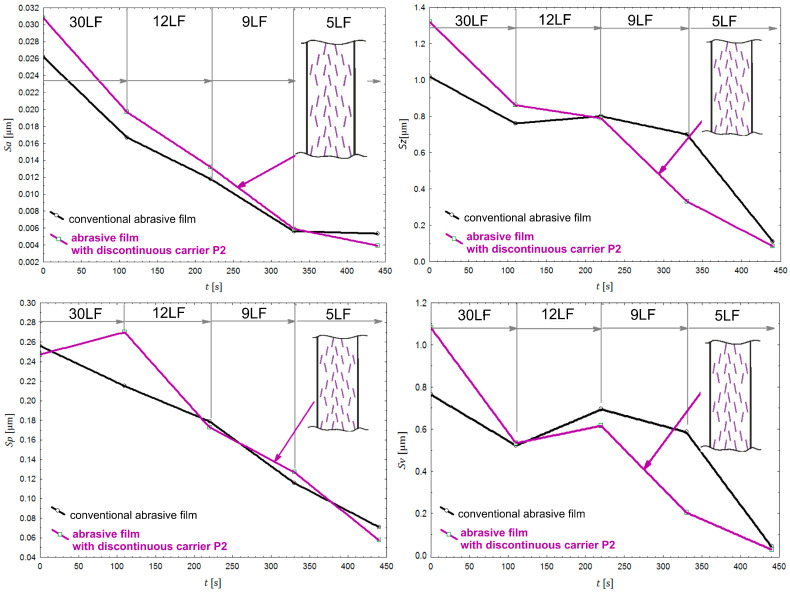
The parameters (Sa, Sz, Sp, and Sv) for assessing surface roughness after the finishing process with abrasive films featuring P2 carrier irregularities (purple color) and conventional abrasive films with nominal grain sizes in micrometers: 30 (30LF), 12 (12LF), 9 (9LF), and 5 (5LF).

When analyzing the results of surface finishing using abrasive films with carrier irregularities in pattern P2 (Figure 12), where additional oscillatory tool motion was applied, focusing on the Sa parameter, it was observed that the film with longitudinal incisions exhibited slightly faster removal of irregularities compared to the conventional abrasive film. 

This phenomenon may be attributed to the additional micro-oscillation of abrasive grains around the irregularities, resulting in faster removal of irregularities from the workpiece material. In the case of machining with additional oscillatory tool motion, significant differences were also evident in the analysis of the Sv and Sz parameters. 

Surfaces machined with abrasive films featuring irregular surfaces showed significantly shallower maximum depths after the smoothing operation with abrasive films with a nominal grain size of 9 μm. However, these differences were not as pronounced as in the case of machining without the use of oscillatory tool motion, which may also be influenced by the nature of the surface irregularities. This suggests that the combination of abrasive films with carrier irregularities and additional oscillatory tool motion has the potential to further improve surface quality by reducing the maximum depths of irregularities. Further investigation into the interaction between the irregular surface characteristics and the oscillatory motion of the tool could provide deeper insights into optimizing the smoothing process for enhanced surface finishing.

Research on tools with carrier irregularities indicates that the distribution of grain peaks and their concentration are not the only factors that affect the quality of a surface processed during surface smoothing. The additional micro-oscillatory motion of abrasive grains around irregularities, which are delicately inclined relative to the direction of lapping film movement, also has a significant influence on machining. This phenomenon leads to faster removal of material irregularities, thus resulting in greater machining efficiency.

## 4. Summary and Conclusions

The research presented in this article introduces innovative designs for abrasive tools aimed at enhancing surface smoothing processes. Through the development of prototypes consisting of non-continuous strips of abrasive films characterized by a discontinuous surface carrier and an abrasive layer, significant advancements in the efficiency and effectiveness of the smoothing process were achieved. This study explored the versatility and efficacy of these prototypes by fabricating four distinct abrasive films with varying nominal grain sizes. The findings highlight the profound impact of carrier irregularities on the surface finishing process, demonstrating notable improvements in material removal efficiency and surface quality. Moreover, the analysis underscores the importance of local abrasive grain displacement trajectories in optimizing material removal processes and ensuring surface quality, offering the following valuable insights for future abrasive tool designs and machining strategies:

- Abrasive films with carrier irregularities in the form of holes result in a significant reduction in surface roughness, while abrasive films with carrier irregularities in the form of longitudinal cuts allow for increased processing efficiency. The trajectory of grain movement along the edges of slanted cuts may be slightly undulated, providing a beneficial effect. In this variation of abrasive film irregularities (pattern P2), the interaction of their edges promotes the formation of longitudinal protrusions, which limits the reduction in the parameter for assessing the surface roughness of the machined surface (Sp) during smoothing with films with grain sizes of 30 and 15 μm. Circular cuts (pattern P1) on the carrier surfaces of abrasive films, on the other hand, allow for the accumulation of larger microchip fragments and enable the formation of a favorable structure of the smoothed surface, as evidenced by reductions in parameters for assessing the roughness of machined surfaces (Sz and Sv).
- Utilizing abrasive tools with carrier irregularities leads to the effective removal of loose grains from the machining zone. The presence of longitudinal irregularities enables faster removal of irregularities from the workpiece material, effectively reducing the risk of deep scratches on surfaces.
- Research has shown that the use of abrasive tools with carrier irregularities limits the depths of scratches on machined surfaces. This results in a significant improvement in finishing quality, which is particularly important for components with high requirements.
- Introducing innovative abrasive tools can contribute to reducing the consumption of materials and natural resources. Lower abrasive consumption means less environmental burden, representing a significant step towards more sustainable production.
- The research findings indicate a clear improvement in the efficiency of surface smoothing processes using abrasive tools with carrier irregularities. This allows for better results to be achieved with reduced time and energy consumption.
- Utilizing abrasive tools with carrier irregularities may lead to a reduction in abrasive consumption. This can lower the costs associated with purchasing and using abrasive materials, positively impacting the profitability of production processes.

## 5. Patents

The developed abrasive films for the process of surface finishing were patented: Kacalak, W., & Tandecka, K. (2014). Abrasive foil for the process of surface micro-smoothing No. P.407465. Exclusive Right No. Pat.232482. Filed 10 March 2014. Assigned to Koszalin University of Technology, Koszalin, PL. International Patent Classification: B24D 11/02, B24D 3/20.

## Figures and Tables

**Table 1:** Machining conditions during the experiments.

Workpiece Material	Chrome steel (40H) (60 HRC)
Pressure roller hardness	50 °Sh
Pressure force	50 N
Tool speed	160 mm/min
Workpiece speed	40 m/min

## Data Availability

The data are contained within the article.
